# Skill and efficiency in Acheulian giant core reduction

**DOI:** 10.1098/rsos.250695

**Published:** 2025-06-25

**Authors:** Coen G. Wilson, Matthew V. Caruana, Bruce Bradley, Andy I. R. Herries

**Affiliations:** ^1^ Department of Archaeology and History, La Trobe University, Melbourne, Victoria, Australia; ^2^ Palaeo-Research Institute, University of Johannesburg Faculty of Humanities, Auckland Park, South Africa; ^3^ Department of Archaeology, University of Exeter, Exeter, UK

**Keywords:** large flake blanks, handaxes, cleavers, core reduction, quartzite

## Abstract

The beginning of the Chibanian age (0.774–0.129 Ma) is characterized by a proliferation of giant core (GC) reduction strategies across the Acheulian world, which were used to produce standardized large flake blanks for shaping large cutting tools (LCTs), such as handaxes and cleavers. Archaeological analyses of GCs have revealed that flaking strategies were often tailored to the lithological and morphological properties of boulders, while experimental research has demonstrated challenges in managing and exploiting their volumes. These insights emphasize the importance of technological skill in reducing GCs efficiently to maximize the production of standardized blanks. However, examining skill in Acheulian contexts has focused on shaping LCTs almost exclusively, while the early phases of these manufacturing chains, including raw material acquisition and blank production, have received less attention. Here, we document the role of technological skill in reducing giant quartzite cores to manufacture large flake blanks through a set of actualistic experiments that contrast expert, intermediate and novice performances. Our results show that knapping expertise is correlated with increased efficiency in exploiting core volume and creating blanks with morphometric features that are ideal for shaping LCTs. We further argue that economizing LCT shaping processes through front-loading time investment in blank production probably involved expert cognition and may have had social implications for skill development in the deep past.

## 1. Introduction

A diverse range of giant core (GC) reduction strategies, including the slab-slice, Kombewa, Victoria West, Tabelbala-Tachenghit and Levallois methods, developed in the Acheulian archaeological record during the transition from the Calabrian (1.8−0.77 Ma) to the Chibanian (774–129 ka) [1–3]. The proliferation of these strategies after approximately 1 Ma has been documented in different regions of the Acheulian world, including the Levant, India, Spain, southern Africa and western Asia, which reflects a significant investment in the use of large flakes (>100 mm in dimension) as blanks for shaping large cutting tools (LCTs, e.g. handaxes and cleavers) [1–12]. The benefits of large flake blanks (LFBs) have been discussed in experimental and archaeological studies alike, which concur that their morphologies can sometimes lessen the technical demands of shaping LCTs [3,4,11–17]. Large flakes have predefined bifacial edges and often only require thinning percussive bulbs and minimal edge modification to achieve desired LCT forms [3,4,16]. Moreover, LFBs can circumvent the need for extensive thinning routines in handaxe manufacture [4,11,12,15–17], widely regarded as one of the most challenging knapping routines in Acheulian toolmaking [18–22].

Few studies have focused on GC reduction specifically, although they have, in some cases, highlighted complex flaking sequences to detach standardized blanks [2,15,17,23,24]. Actualistic experiments have further demonstrated that organized knapping strategies are required to effectively exploit GC volumes [17,25–28]. Therefore, the ability to manage GC reduction strategies, while maximizing LFB production likely required a high degree of technical knowledge and practical experience [11,17,24,27,29–31]. Yet, previous research investigating technological skill in Acheulian contexts has mostly focused on LCT shaping processes, which has provided important insight into requisite motor-cognitive capacities and social scaffolding [13,19,20,32–34]. Examining how technological skill manifests across all phases of LCT production is also important for understanding the significance of blank acquisition and its impact on subsequent manufacturing stages [11,30,35,36].

While experimental research replicating GC reduction has rarely addressed the role of technological skill directly, the concept of efficiency (often measured as the number of LFBs obtained) has been used to assess the rates of LCT blank production [17,25–27]. In fact, measuring efficiency in core reduction remains a focal point of quantitative archaeological research examining evolutionary trends in lithic technologies [37–40]. Although efficiency can be defined and measured in different ways, its maximization in blank production generally correlates with highly skilled knapping performances [20,38,39,41–45]. Developing perspectives on how technological skill in GC reduction impacts the production of LFBs can therefore provide insight into the economics of Acheulian toolmaking strategies [16].

To investigate the effects of skill on GC reduction, we present the results of actualistic experiments comparing expert, intermediate and novice performances, along with core and blank products. Comparative analyses focus on efficiency, defined as the average number of strikes needed to produce a set of LFBs, as well as the amount of volume retained in blanks relative to its loss in cores. Productivity is also measured, defined as the number of useable LFBs produced and their morphometric features. Expert performance in this context should theoretically result in a higher number of standardized flake blanks successfully converted from core volume, while minimizing energy output [42,45,46].

Developing the technical skills to reduce GCs efficiently would have afforded flexibility in LCT manufacturing chains, specifically front-loading time investment in blank acquisition to alleviate the challenges of shaping and thinning routines. From a cognitive perspective, modifying manufacturing chains in this manner would have likely involved the retrieval and recombination of procedural memories associated with expert cognition [30,47–49]. Information related to the order of LCT production procedures could be recalled and arranged in ways that anticipated constraints, such as heterogenous raw material lithologies or a need to expedite tool production. Understanding how GC reduction modifies LCT manufacturing processes is therefore important for expanding perspectives on the flexibility and planning depth that structured Acheulian technological systems.

## 2. Background

### 2.1. The significance of the Acheulian giant core reduction

The procurement and use of large flake blanks, over >100 mm in maximal dimension (see [1] for definition), played an important role in Acheulian LCT production strategies since their origins ≥1.7 Ma [50–53]. Early Acheulian sites (1.7–1.0 Ma) rarely contain GCs and few reveal complex reduction systematics [52,54–56]. Sometime around or after 1 Ma, organized GC reduction methods proliferate within ‘Large Flake Acheulian’ industries, defined by the prevalence of LFB use in LCT production [4].

Sharon’s [1–4,57] investigation of Large Flake Acheulian assemblages has revealed a diverse set of GC reduction methods, which had a significant impact on LCT manufacturing chains. He suggested that ‘[t]he ability to predetermine blank shape prior to its detachment from the parent GC, and the knowledge that guided the blank selection process, enabled the makers of large flakes to produce blanks of desirable shape that needed almost no additional shaping work’ [4, p. 228]. Blank production and selection can therefore alter LCT reduction sequences, and in some cases, mitigate skill-demanding shaping processes [11,13]. For example, recent research has demonstrated that the production of handaxes at the later Acheulian site of Amanzi Springs (approx. 534–390 ka; [58,59]) was often hindered by the structural qualities of local quartzites [12,22,59]. Acheulian knappers frequently failed to thin handaxes sufficiently, which led to their early discard. Wilson *et al.* [12] recently identified a trend in the chronostratigraphic sequence of the Area 2 spring eye (approx. 534≤408 ka; [59]), where the use of LFBs increased through time, which correlated with progressively thinner handaxes. This trend demonstrates the ability of Amanzi Springs knappers to modify handaxe manufacturing chains, in which front-loading time investment in LFB production significantly lessened the technical demands of handaxe thinning.

While LFB use economized LCT production in some instances, GC reduction presents challenges that can hinder flaking mechanics and planned flaking sequences. In general, GC reduction requires anticipation in terms of strategies that can be successfully applied to the morphology of raw material packages [4,27]. However, boulders over approximately 50 kg are not easily rotated repeatedly, and thus exploring their geometry can be cumbersome [11: see below]. Furthermore, knappers are sometimes required to stabilize boulders through supports, or wedges, to exploit flaking surfaces [27]. Finally, initiating flake detachments, specifically the opening flake (*éclat entame*), can be challenging due to the toughness of raw materials or lack of appropriate flaking angles on boulders [26,27,60].

Given these difficulties, efficiently reducing GCs is important if the time investment is to outweigh the required energy output. While we acknowledge, such factors were not necessarily the main drivers of lithic production in the deep past, the role of technological skill in this process cannot be underestimated, albeit this issue lacks attention in skill-focused, Acheulian research. Nonetheless, Stout’s [35,36] exploration of stone-knapping skill development in Papua New Guinea emphasized the expertise required to select quality raw materials worth transporting for lithic reduction. Winton’s [13] actualistic experiments documenting handaxe shaping amongst expert and novice knappers also noted the importance of blank selection and use, specifically that elongated and thin cobble blanks bypassed the need for extensive shaping and thinning. Holdaway and Douglass [61] further summarized ethnographic accounts of blade and knife-making amongst Australian Indigenous communities that also consistently highlighted the importance of blank selection. These ethnographic and experimental reports demonstrate that technological skill plays an important role in the early phases of lithic manufacturing chains. Although testing the effects of skill in the deep past presents challenges largely relying on measurements and descriptions of artefact attributes [20,32,33,45,62].

### 2.2. Measuring skill through efficiency and productivity

Examining technological skills in archaeological research is important for interpreting variation in lithic assemblages, as well as understanding evolutionary trends in blank production strategies [16,38–40,42,63,64]. Inferring skill through the analysis of lithic artefacts has largely been operationalized through estimations of complexity in lithic manufacturing procedures [65,66], the frequency of technical mistakes [22,41,42,59,62], or standardization of artefact forms [43,46,67].

Comparing skills in core reduction specifically has been evaluated through both qualitative and quantitative assessments, which are further supported by experimental research [41,42,45,63,68–70]. Qualitative indicators of skilled performance include the frequencies of step and hinge fractures on flaking surfaces, battering on platforms and unsystematic flaking sequences [41,42,45]. Quantitative methods typically involve some measure of ‘efficiency’ through indices that compare features of cores and blank products. For example, initial core mass, or the mass of blanks, relative to the number or cutting-edge length of blanks are variables often used to calculate the rate of core volume converted into a functional tool quality [32,38,42,44,68,69,71]. Other efficiency measures compare the amount of volume in exhausted cores as a proxy for waste [37,45,69], or the number of percussive strikes per flakes produced, which represents energy expenditure [37,72,73]. The important concept here is that knapping expertise should increase the conversion of core volume into desired blank types while minimizing core volume loss and energy expense [38,42,72].

The confluence of skilled core reduction and efficiency is thus important for interpreting the significance of GC technologies in LCT manufacturing chains. Experimental studies replicating GC reduction strategies have provided insights into efficiency, mostly measured through the number of useable LFBs produced, as well as their morphological features [2,25–28]. For instance, Jones [25] reduced a boulder of approximately 13 kg in trial-and-error experiments and successfully detached 5–10 LFBs, while Toth [26] produced 97 LFBs from 20 giant basalt cores over a 5 h period. Qualitative observations of GC reduction processes have also highlighted the need for preparation and planning to efficiently produce LFBs [1,2,11,17,24–28,74,75]. Madsen and Goren-Inbar [27] found that GCs required preparation of flaking surfaces (i.e. managing flake scar ridges and convexities) before useable LFBs could be successfully detached. After preparing surfaces, the experimental knapper was able to produce between 5 and over 30 LFBs from cores ranging up to approximately 200 kg, alluding to the role of technological skill in maximizing blank products. The benefits of preparing LFB detachments were also recently highlighted in Wilson *et al.* [17], who documented a high frequency of core rotation and margin trimming to shape flaking surfaces in expert GC reduction. As such, high levels of efficiency in GC reduction are likely dependent on technological skill, although this issue has yet to be rigorously examined.

### 2.3. Modifying LCT manufacturing chains and the expert mind

Another important aspect of the proliferation of GC reduction in Acheulian technological systems is the implications of modifying LCT manufacturing chains through the use of LFBs and associated cognitive processes. Given that many Acheulian GC reduction methods portray formalized flaking sequences, they likely relied upon procedural memory to execute successfully [48,49,76]. Wynn *et al.* [48,49] have recently described the expert cognition model to conceptualize the evolution of technological skill in relation to the complexity of lithic production systems in the human past. In this context, expert cognition involves the retrieval of procedural memories, represented as ‘chunks of information’ that are inter-related and can be sequentially organized to guide actions [48,49,66,76]. These information chunks are similar to Perrault *et al*.’s [66] concept of ‘procedural units’, which they define as independent stages of lithic production systems (e.g. raw material acquisition or core preparation stages) (cf. [65]). Each procedural unit is cognitively represented by memories of technical actions that relate to sub-goals structuring these stages, which can be interlinked into manufacturing chains. Cognitively speaking, the retrieval and arrangement of procedural units when undertaking tasks is managed by long-term working memory, where well-developed ‘how-to information’ can be recalled to guide technical actions [76]. The way in which Acheulian knappers memorized reduction procedures, recalled them through focused practice and developed technical skills in biomechanical movements, all support the development of expertise [47–49,76].

Moreover, Muller *et al.* [39] suggest that while the number of procedural units can be used to measure the complexity of lithic reduction sequences, understanding their hierarchical relationships is also important for measuring expertise. For example, they demonstrate that bipolar knapping involves only two, hierarchically structured cognitive processes involving flake production and recognizing suitable platforms for further removals. Discoidal reduction, on the other hand, involves four hierarchical processes, including discoidal flaking, establishing bifacial hemispheres, maintaining surface convexities and preparing dihedral platforms [39]. Not only are the number of steps to reduce discoidal cores more numerous when compared with bipolar reduction, but also the hierarchical structure of maintaining surface convexities and preparing platforms reflects the skill required to extend the use-life of a discoidal core. Thus, the order in which procedural units are carried out to achieve specific sub-goals in core reduction is important for tracing skill and complexity throughout lithic production processes.

Further to this point, procedural units can also be recombined in novel ways, which is sometimes referred to as generativity or nested recursion [66,77,78]. This ability to break apart information chunks stored in procedural memories and recombine them in hierarchically structured ways that may improve the success of tasks was likely important for overcoming constraints in lithic production strategies [66]. In Acheulian contexts, procedural units structuring LCT manufacturing chains were modified to increase efficiency and anticipate known challenges, including raw material constraints on LCT shaping and thinning [14,15,17,22,59]. As such, understanding the confluence of skill and cognition in GC reduction for exploiting the benefits of LFB use in manufacturing can further highlight planning depth in Acheulian technological systems.

## 3. Material and methods

### 3.1. Material collection locations

**Figure 1.**
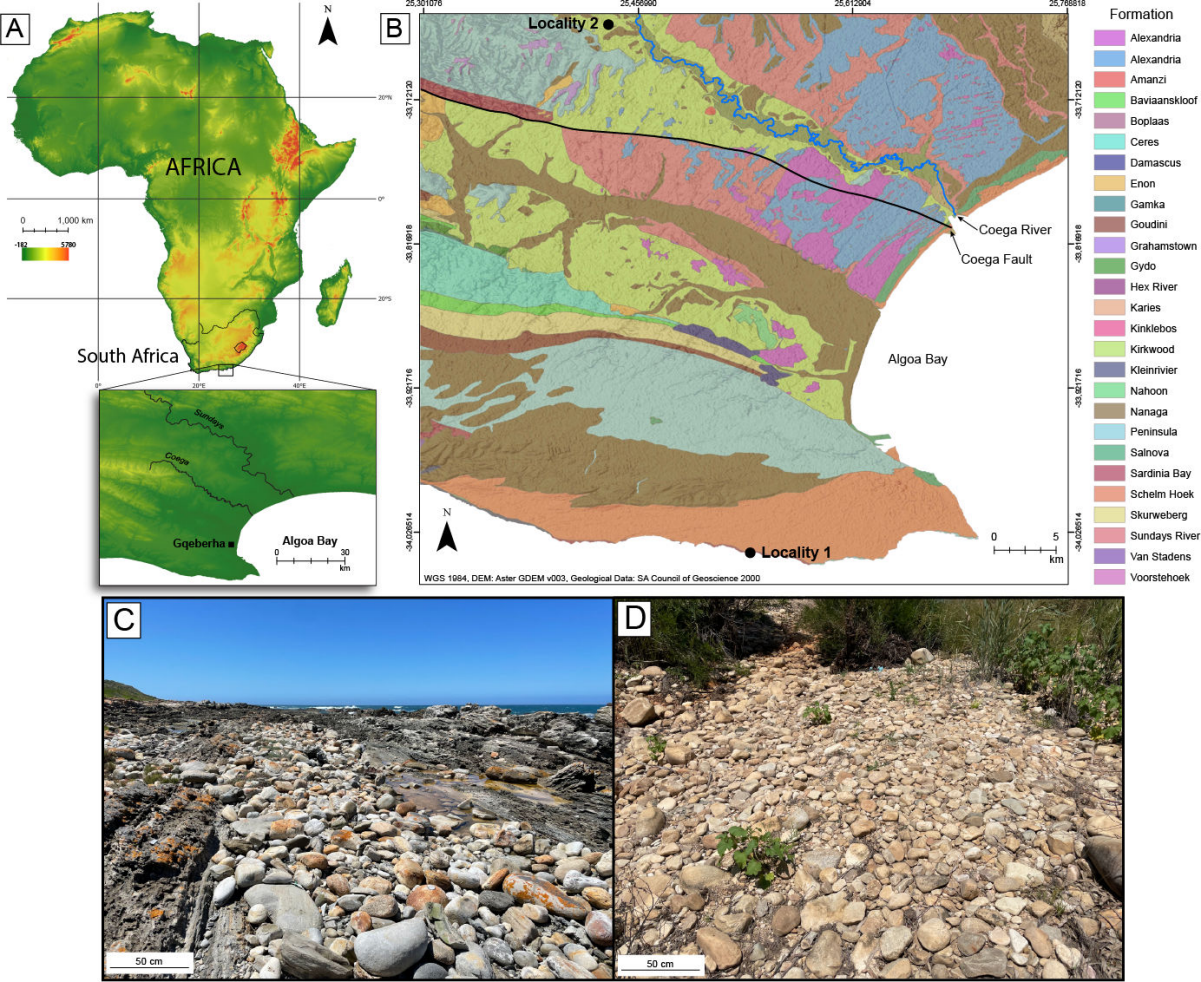
(A) Location of raw material collection area in relation to South Africa. (B) 1:50 k geological map (formations listed alphabetically) of the Nelson Mandela Bay region showing raw material collection localities. (C) Locality 1, beach conglomerate accumulation. (D) Locality 2, Coega alluvial terrace exposure. Adapted from Wilson *et al.* [17].

The quartzite boulders used in the actualistic experiments described below are all from the Table Mountain Group (TMG) and were collected from Nelson Mandela Bay (Eastern Cape Province, South Africa), following survey and selection procedures outlined in Wilson *et al.* [17]. Boulders were collected from two previously identified sources: Locality 1 (beach conglomerate accumulation), and Locality 2 (Coega alluvial terrace exposure), which contain an array of quartzite lithologies and range in size from pebbles to boulders (figure 1). Locality 1 quartzites are composed of fine- to medium-grained texture that are blue-to-dark blue in colour, and have a thin, white cortex, whereas Locality 2 quartzites are composed of moderately sorted, coarse- to very coarse-grained texture that are grey to light grey in colour with a thick, dark orange cortex [17]. Although the quartzites used in this experiment are from two different geographical locations, they share similar mechanical characteristics and are comparable in their fracture properties when tested under hard hammer percussion. In addition, recent experimental results from Wilson *et al.* [17] showed that the two different quartzite qualities are equally as efficient in producing LFBs of uniform dimensions.

Boulder selection was informed by previous experimental and archaeology data of GC geometric attributes to ensure morphological consistency [17,25–28,59,79]. This included selecting boulders that exhibited a naturally flat upper and/or base cortical surface to serve as a striking platform, a slanting or ridged section which created an acute angle to the base or appropriate edge angles for flake detachment around the boulder’s circumference, and the presence of natural impact cones [17]. These attributes afforded participants at least one entry point into the volume of boulders for initiating flake removal sequences. A range of quartzite hammerstones differing in size, morphology, surface textures and weights were also collected at each raw material locality. Hammerstone selection was based on their manipulation potential, such that they could be held safely and comfortably in one or two hands, allowing enough mass to protrude from the gripping hand(s) so they were not vulnerable when striking the core, as well as surface features including smooth textures with impact cones.

### 3.2. Experimental protocols

Our actualistic experiments aimed to investigate the effects of knapping skill on GC reduction sequences by comparing performances and LFB products. Raw materials were restricted to quartzites to control for lithological properties affecting fracture mechanics. Participants were divided into three respective skill groups based on their stone-knapping experience. Novice participants (*n* = 2) had no prior knowledge or experience in stone tool replication, while the intermediates (*n* = 2) had a good theoretical and working knowledge of stone tool manufacture techniques but infrequently engaged in stone knapping. One expert knapper had over approximately 50 years of experience replicating stone tools and an extensive theoretical knowledge how to produce a range of different lithic technologies. The experts and intermediates previously engaged with knapping stationary cores, while novices had no experience in such techniques. Furthermore, the expert has knapped GCs for over 20 years. All participants provided written informed consent prior to the enrolment in this study, under protocols approved by La Trobe University. Expert knapping data were collected by C.G.W. and M.V.C. at the University of Johannesburg (Gauteng Province, South Africa) and the novice and intermediate knapping data were collected by C.G.W. and M.V.C. in Gqeberha (Eastern Cape Province, South Africa).

Before the novice participants reduced their cores, they received brief instructions on stone-knapping basics, which discussed core rotation and stabilization, hammerstone selection and striking motions for freehand percussion, in an effort to prevent injury. Both novice and intermediate participants viewed a video recording of the expert participant reducing a GC, without verbal instructions. We assume that neophyte knappers in Acheulian groups would have had some exposure to socially structure knapping practices and thus were not completely naïve to such processes in the deep past. In addition, they were also able to inspect and handle examples of LFBs produced by the expert participant to see the products. All participants were instructed to reduce quartzite boulders with the intention to produce LFBs (>100 mm in dimension); however, they saw fit, without adhering to any specific core reduction strategy. Reduction experiments were stopped when cores were either exhausted or when producing LFBs was not possible due to steep core convexities, obtuse platform angles or raw material flaws. Novice participants were stopped at the approximately 15 min mark if they had not detached any LFBs.

GC reduction experiments were conducted on large canvas tarpaulin, to ensure all flaked pieces, debitage and debris were recovered. Participants either sat or kneeled with the core horizontally resting upon the ground in front of them (electronic supplementary material, figure S1). The expert participant was allocated five quartzite boulders to reduce (two from the beach conglomerate and three from the alluvial terrace), whereas the intermediate and novice participants were allocated four quartzite boulders each, two from the collection localities mentioned above. Participants were allowed to move and rotate the GCs freely, using additional hammerstones as wedges to angle and support cores. All knapping participants were presented with a selection of quartzite hammerstones, ranging between 0.645 and 9.980 kg, during each reduction experiment. Hammerstone selection and switching was recorded for each strike, along with the number of hands needed to manipulate them in percussive actions.

### 3.3. Metric variables

All quartzite boulders and hammerstones were weighed to the nearest 0.01 g, measured in maximum length, width and thickness (mm), photographed on all sides and three-dimensionally scanned before reduction. This process was repeated for all exhausted cores, LFBs and hammerstones after the experiments. Three-dimensional scanning was completed using an Artec Space Spider scanner and processed using the Artec Studio 15 Professional software, which produced water-tight meshes saved as .WRL files. These meshes were then imported into MeshLab (v.2020.07) where surface area (mm^2^) and volume (mm^3^) measurements were collected. Two-dimensional shape variability in LFBs was investigated using elongation (length/width) and refinement (width/thickness) ratios [80]. Categorical variables for LFBs were also collected, included the flake blank type (i.e. entame, side, end and corner struck categories), dorsal cortex presence, longitudinal splits or transverse fractures, platform and termination type, plan and profile shape and dorsal scar count and orientation [1–3,81–83].

Each GC reduction event was timed and video recorded to assess knapping strategies and gestures. Percussive strikes were recorded as three separate categories, including unsuccessful strikes (no flake detached), debitage strikes (detaching flakes <100 mm in dimension) and LFBs strikes (detaching flakes ≥ 100 mm in dimension). The average strike rate was then calculated by dividing the number of total strikes by the total reduction time in minutes. Core rotational patterns were also recorded, defined as a change in the core position using a turn or flip by the participant [84]. Due to the large sizes of GCs, and the heavy force often required to detach an LFBs, there is usually movement in the core’s position, caused by percussive strikes. As such, rotational data were only counted when the participant actively rotated the core more than 90° on its vertical axis or flipped the core 180° to the opposing face along the horizontal axis of the blank. Diacritical descriptions of reduction sequences across skill groups were used to investigate flaking strategies and volume management in exhausted or abandoned GCs [85,86].

### 3.4. Efficiency and productivity variables

Efficiency and productivity indices used to compare skill groups were derived from archaeological studies examining core reduction processes, including Prasciunas [68], Jennings *et al.* [69], Putt [37] and Wilson *et al.* [45]. In this study, we followed Putt [37] in differentiating variables that represent aspects of efficiency and productivity. Here, efficiency is defined as both the energy expended (i.e. the average number of percussive strikes) during the GC reduction process, as well as the retention of core volume in blanks. Efficient core reduction is thus represented by a relative low number of strikes that result in a high number of flake blanks, which maximize the conversion of core volume. Productivity is defined as the total number of LFBs produced, as well as desirable morphometric features (i.e. elongated and thin flakes) that are ideal for shaping LCTs. Disentangled, these concepts provide a more accurate quantitative assessment of how skill impacts both the processes of GC reduction (i.e. efficiency) and LFB production (i.e. productivity).

Efficiency variables include:

— Percentage of GC volume lost during reduction (initial core volume-final core volume/initial core volume*100 [mm^3^ mm^−3^])
— GC volume converted into large flake blanks (LFB count/initial core volume [*n* cm^−3^])
— GC volume converted into LFB volume (LFB volume/initial core volume [cm^3^ cm^−3^])
— Average strikes per minute (strike count/total time in minutes [*n* min^−1^])

Productivity variables include:

— Total number of LFBs per GC reduction event (*n*)
— Elongation in LFBs (length/width [mm mm^−1^])
— Refinement in LFBs (width/thickness [mm mm^−1^])

Due to the non-parametric nature of the recorded data and small sample sizes in some cases, Mann–Whitney *U* tests (α = 0.05) with Monte Carlo simulations were used to compare all metric, efficiency and productivity variables for GCs and LFBs across skill groups in SPSS v.29. All boxplot and regression scatterplot graphs were produced in PAST 4.13.

## 4. Results

### 4.1. Reduction experiments

**Figure 2.**
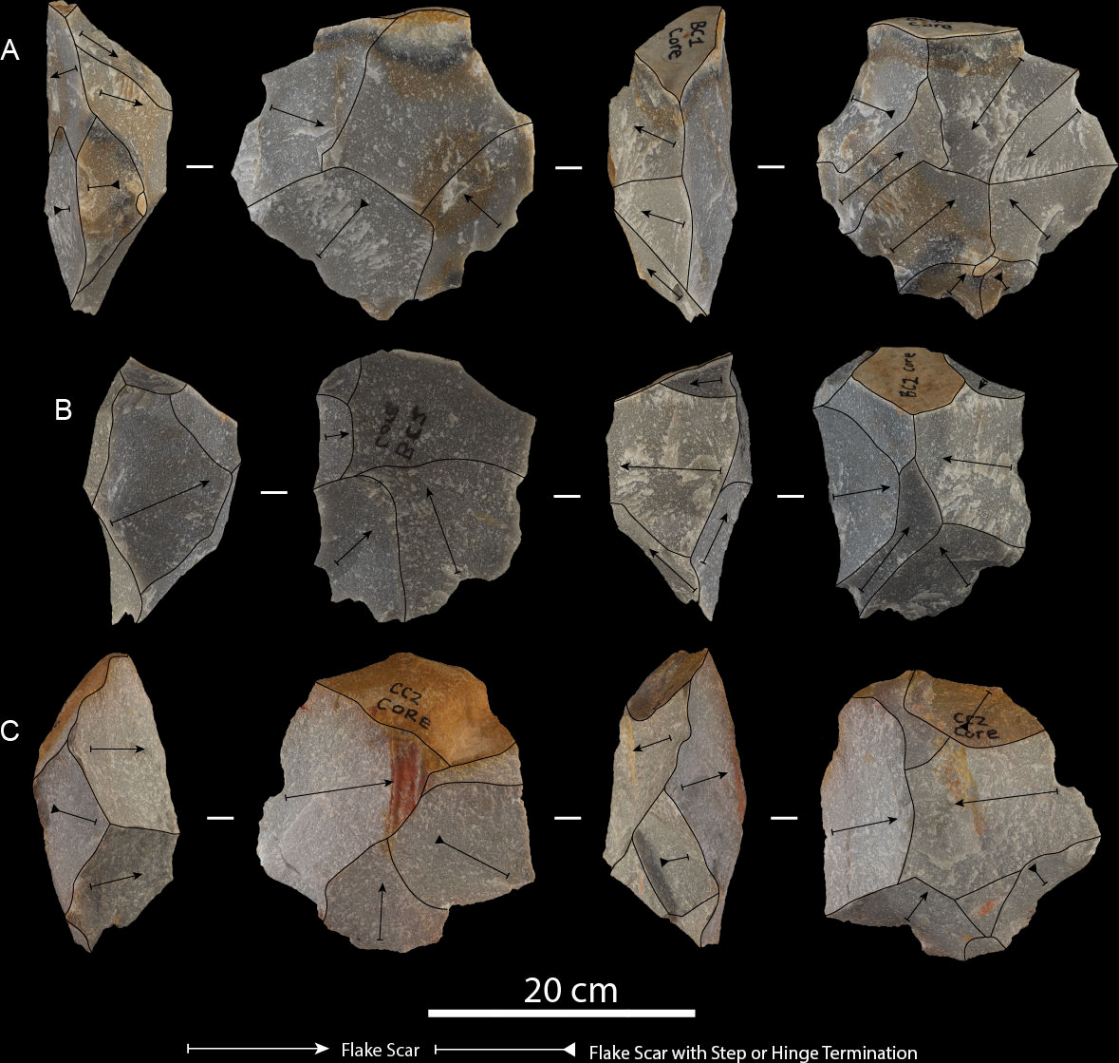
Expert cores: (A) Beach conglomerate core 1, (B) Beach conglomerate core 2 and (C) Coega terrace core 2.

The expert participant reduced five quartzite boulders, ranging in maximum length from 290 to 370 mm and weighing between approximately 10 and 24 kg (electronic supplementary material, table S1). Hammerstone strike rates ranged between 28 and 54, with an average of 37.6 strikes per core reduction sequence (electronic supplementary material, tables S1 and S2). Of the total number of hammerstone strikes, 53.1% (*n* = 100) were unsuccessful in detaching a flake product (electronic supplementary material, table S2). On average, three hammerstones were used per reduction sequence, which was always held with one hand, using the other hand to stabilize the GC (electronic supplementary material, table S2). In terms of core reduction strategies, the expert participant mostly employed a discoidal method through bifacial alternating flaking (*n* = 4; 80%) and the rotation-to-strike ratio averaged 3.4 per reduction sequence (figure 2; electronic supplementary material, table S1). Knapping gestures and core movements of the expert participant were consistent in all five core reduction experiments. Exploitation of cores focused on establishing adequate platforms around the circumference of the GC, before beginning a sequence of invasive flakes directed towards the centre mass to maximize flaking surface dimensions (figure 2). The expert produced a total of 60 LFBs in 58.77 min, which exceeds all other skill groups in terms of production rates (table 1; electronic supplementary material, table S2). Flaking ceased due to core exhaustion, poor surface convexities for LFB production and obtuse platform angles. Comprehensive knapping descriptions are provided for each expert GC reduction in Wilson *et al.* [17].

**Table 1.** Descriptive statistics for large flake blanks (s.e. = standard error; CV = coefficient of variation; s.d. = standard deviation).

	length (mm)	width (mm)	thickness (mm)	m	surface area (mm^2^)	volume (mm^3^)	length/width	width/thickness
expert (60)								
mean	159.74	134.43	47.17	1016.5	41 279.62	384 428.30	1.25	2.98
s.e.	5.16	5.05	1.83	103.01	2535.58	38 992.99	0.04	0.12
median	157.68	128.38	44.72	805	36 569.93	302 924.00	1.23	2.81
CV	25.02	29.10	30.11	78.49	47.57	78.56	28.30	32.15
s.d.	39.97	39.13	14.20	797.91	19 640.54	302 038.40	0.35	0.96
min	97.10	59.04	21.88	165	12 220.56	61 550.47	0.50	1.63
max	236.29	247.52	78.60	4375	109 302.50	1 661 387.00	2.09	6.96
intermediate (63)								
mean	137.36	142.74	43.58	899.57	21 645.91	358 472.70	1.02	3.45
s.e.	4.31	5.60	2.01	98.43	1849.41	39 557.87	0.04	0.12
median	139.08	128.78	42.76	610	18 388.95	250 397.20	0.97	3.24
CV	24.94	31.18	36.61	86.85	67.81	87.58	32.03	28.24
s.d.	34.27	44.51	15.95	781.33	14 679.29	313 980.80	0.32	0.97
min.	73.95	64.66	19.76	150	5369.61	58 056.66	0.40	1.44
max.	232.85	271.89	94.53	4715	66 485.21	1 892 910.00	2.10	7.20
novice (6)								
mean	90.73	120.55	28.91	334.16	22 495.43	128 697.90	0.78	4.32
s.e.	4.50	10.18	2.51	52.73	3310.38	20 415.05	0.07	0.49
median	87.57	109.31	27.88	320	20 918.14	121 165.30	0.81	4.48
CV	12.17	20.68	21.32	38.65	36.04	38.85	22.48	28.19
s.d.	11.04	24.93	6.16	129.16	8108.75	50 006.46	0.17	1.22
min	77.95	101.22	22.26	185	14 472.44	70 249.48	0.52	2.59
max	109.90	164.98	39.12	530	33 977.17	199 575.60	0.97	6.22

**Figure 3.**
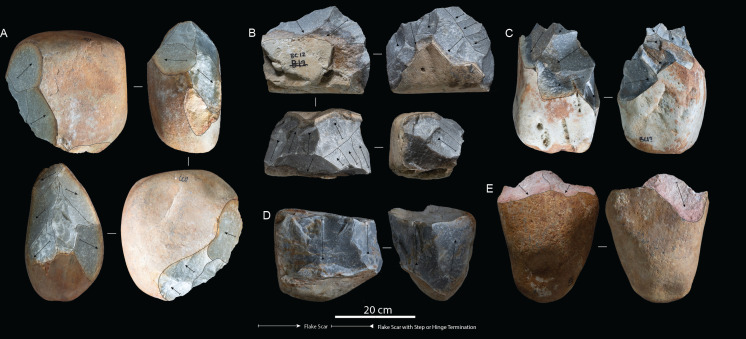
Intermediate cores: (A) Coega terrace core 11, (B) Beach conglomerate core 12, (C) Beach conglomerate core 13, (D) Beach conglomerate core 11 and (E) Coega terrace core 9.

Intermediate participants reduced eight quartzite boulders, ranging in maximum length from 320 to 450 mm and weighing between approximately 17 and 31 kg (electronic supplementary material, table S1). Hammerstone strike rates ranged between 54 and 129, with an average of 82.1 strikes per core reduction sequence (electronic supplementary material, tables S1 and S2). Of the total number of hammerstone strikes, 76.7% (*n* = 505) were unsuccessful in detaching a flake product (electronic supplementary material, table S2). On average, five hammerstones were used per reduction sequence, which was predominantly held with one hand 68.6 (*n* = 451), using the other hand to stabilize the GC (electronic supplementary material, table S2). In terms of GC reduction strategies, the intermediate participants used unifacial (*n* = 3; 37%) and bifacial, continuous (*n* = 3; 37%), alternate (*n* = 1; 13%) and alternating (*n* = 1; 13%) flaking, which did not exploit the full core volume (figure 3; electronic supplementary material, table S1). The rotation-to-strike ratio was an average of 9.6 per reduction sequence (electronic supplementary material, table S1). Intermediate participants detached a total of 63 LFBs in 104.03 min (table 1; electronic supplementary material, table S2). Similar to the expert participant, intermediates were able to successfully identify appropriate edge angles for the detachment of a core opening flake (*éclat entame*). This initial LFB would require several repeated strikes, with the participants experimenting with different hammerstone weights, grips and percussion angles during this initial stage of reduction. Once detached, knapping strategies would begin to follow in a similar manner to the expert sequences, alternating between core faces to detach LFBs and often requiring repeated strikes to detach flakes.

**Figure 4.**
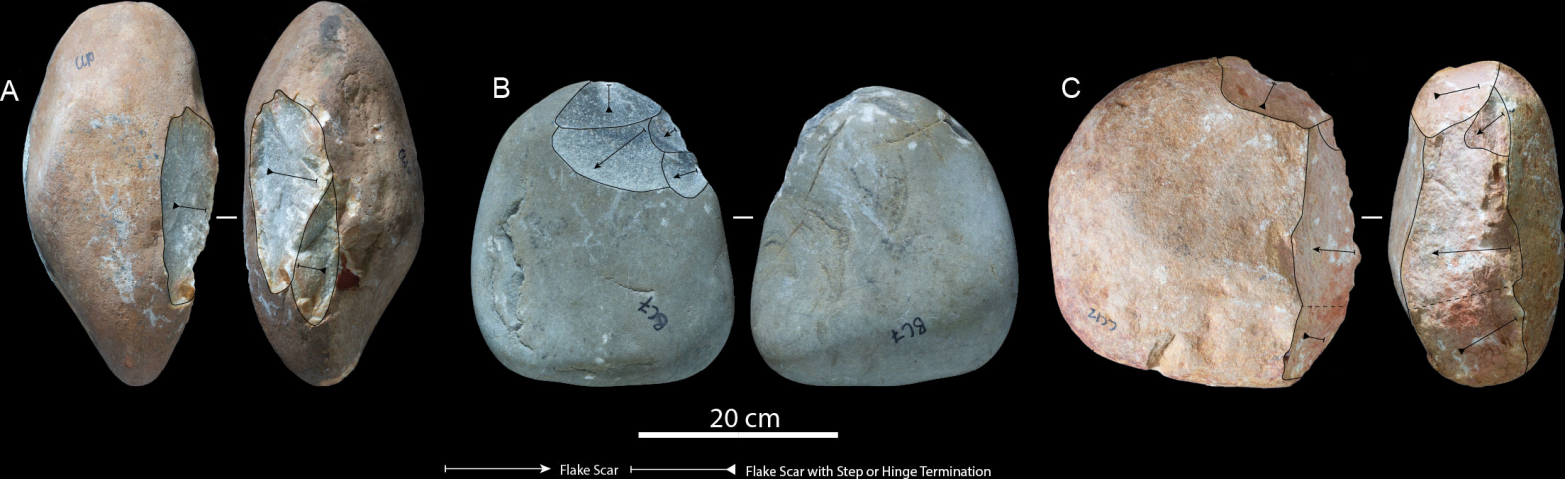
Novice cores: (A) Coega terrace core 10, (B) Beach conglomerate core 7 and (C) Coega terrace core 12.

Novice participants reduced eight quartzite boulders, ranging in maximum length from 280 to 360 mm and weighing between approximately 9 and 17 kg, although were unable to detach LFBs on four of the GCs (electronic supplementary material, table S1). Hammerstone strike rates ranged between 37 and 137 with an average of 84.7 strikes per core reduction sequence (electronic supplementary material, table S2). Of the total number of hammerstone strikes, 91.5% (*n* = 620) were unsuccessful in detaching flakes, with an average of 77.5 unsuccessful strikes per core reduction (electronic supplementary material, table S2). On average, 2.2 hammerstones were used per reduction sequence, which were held with two hands 84.2% (*n* = 571), using their body or another hammerstone to stabilize GCs. Examining the core reduction strategies, the novice participants employed short, unifacial, unipolar flaking techniques (figure 4; electronic supplementary material, table S1). The rotation-to-strike ratio was an average of 6.7 per reduction sequence, and novices produced a total of 6 LFBs in 88.59 min (table 1; electronic supplementary material, table S2). Initiating a core opening flake (*éclat entame*) was a significant issue for novice participants, often due to the inability to identify a suitable edge angles or placement of percussive strikes. Additional hammerstones were often used as wedges to stabilize GCs during reduction. However, GCs would constantly shift after each strike, creating a discontinuous reduction sequence where the novice participants constantly adjusted cores after each strike.

### 4.2. Giant cores and large flakes

**Figure 5.**
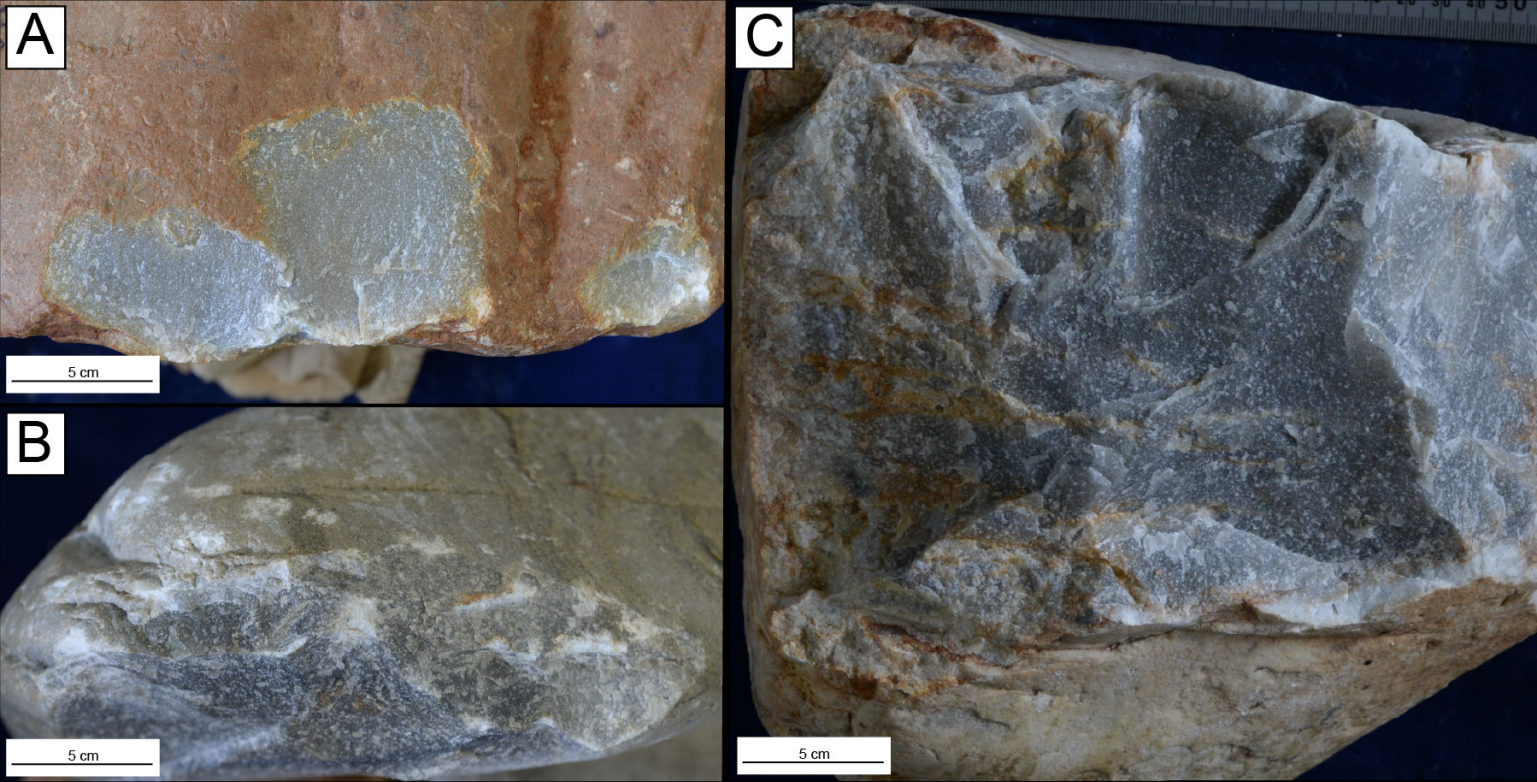
Core damage: (A) non-invasive, shallow flake scars (CTC6), (B) repetitive percussive damage (BCC7), and (C) step fracture and edge crushing due to internal raw material flaws (BCC11).

All descriptive and metric variables recorded for GCs, LFBs and hammerstones are reported in tables 1 and 2; electronic supplementary material, table S4. The average volume loss in GCs across skill groups shows a linear relationship correlated with expertise, where the expert reduced 69.8%, intermediates reduced 38.47% and novices reduced 4.61% of total core volume (electronic supplementary material, table S1). The expert participant produced one unifacial and four discoidal cores (figure 2). Minimal platform crushing was observed on these cores and step/hinge fracturing was isolated to their margins, which indicates a high accuracy and control of percussive strikes. Intermediate GCs showed the most variation in reduction strategies and final core morphologies when compared with expert and novice participants, including five bifacial and three unifacial cores (figure 3). Intermediate cores show partial exploitation of core volumes, and flaking strategies were used based on the overall morphology of raw material packages and suitable platform angles. When compared with the expert sample, the intermediate cores display crushing on platforms and more step and hinge fractures on flaking surfaces. Novice cores were all unifacial and display shallow, non-invasive flake scars along core margins (figure 4). These cores further show pervasive platform crushing and battering, along with high frequencies of stacked step and hinge fractures on flaking surfaces (figure 5).

**Table 2.** Descriptive variables were used for the analysis of large flake blanks. Blank type: (1) corner struck, (2) end struck, (3) side struck, (4) entame. Platform type: (1) cortical, (2) plain, (3) dihedral, (4) crushed. Flake plan shape: (1) convergent, (2) expanding, (3) parallel, (4) round. Flake edge profile shape: (1) straight, (2) curved, (3) twisted. Dorsal scar pattern: (1) unidirectional, (2) bidirectional, (3) sub-radial, (4) radial, (5) cortex. M = mean.

expertise	core *n*	LFB *n*	cortex *n*/%	flake scars (M)	split blank *n*/%	blank type *n*/%				platform type *n*/%				plan shape *n*/%				profile shape *n*/%			dorsal scar pattern *n*/%				
						1	2	3	4	1	2	3	4	1	2	3	4	1	2	3	1	2	3	4	5
expert	5	60	56/ 93	1.5	13/ 22	5/ 8	42/ 70	8/ 13	5/ 8	11/ 18	38/ 63	11/ 18	—	20/ 33	27/ 45	13/ 21	—	46/ 76	9/ 15	5/ 8	26/ 43	7/ 11	13/ 21	3/ 5	11/ 18
intermediate	8	63	57/ 90	1.3	18/ 28	2/ 3	42/ 66	11/ 17	8/ 12	22/ 35	38/ 60	—	3/ 5	12/ 19	38/ 60	12/ 19	1/ 1	41/ 65	22/ 35	—	39/ 62	1/ 1	7/ 11	—	16/ 25
novice	8	6	6/ 100	0.5	1/ 16	—	3/ 50	—	3/ 50	6/ 100	—	—	—	2/ 33	4/ 67	—	—	4/ 66	1/ 16	1/ 16	3/ 50	—	—	—	3/ 50
	21	129																							

**Figure 6.**
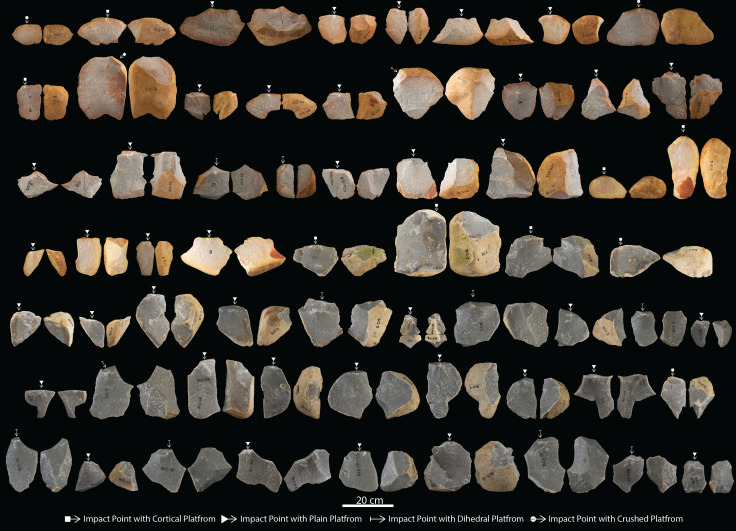
Expert large flake blanks detached from cores.

**Figure 7.**
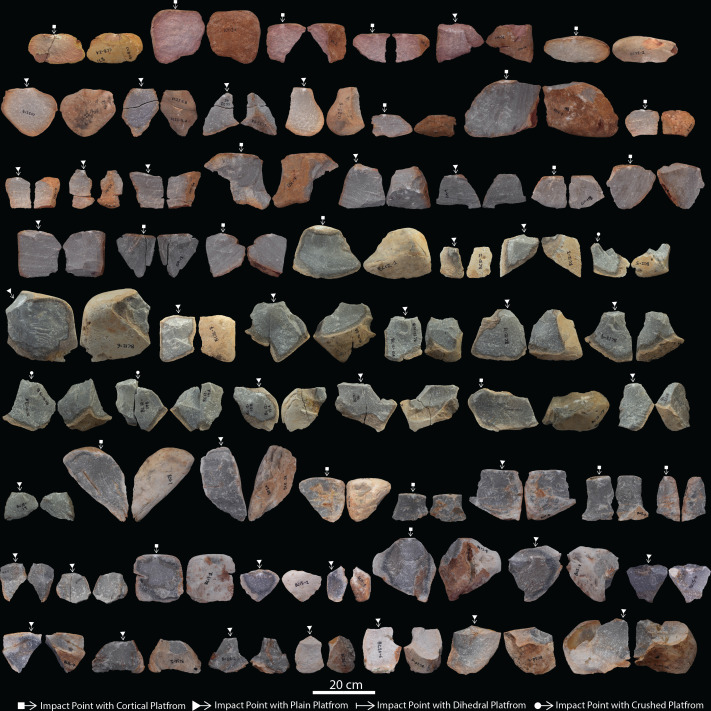
Intermediate large flake blanks detached from cores.

**Figure 8.**
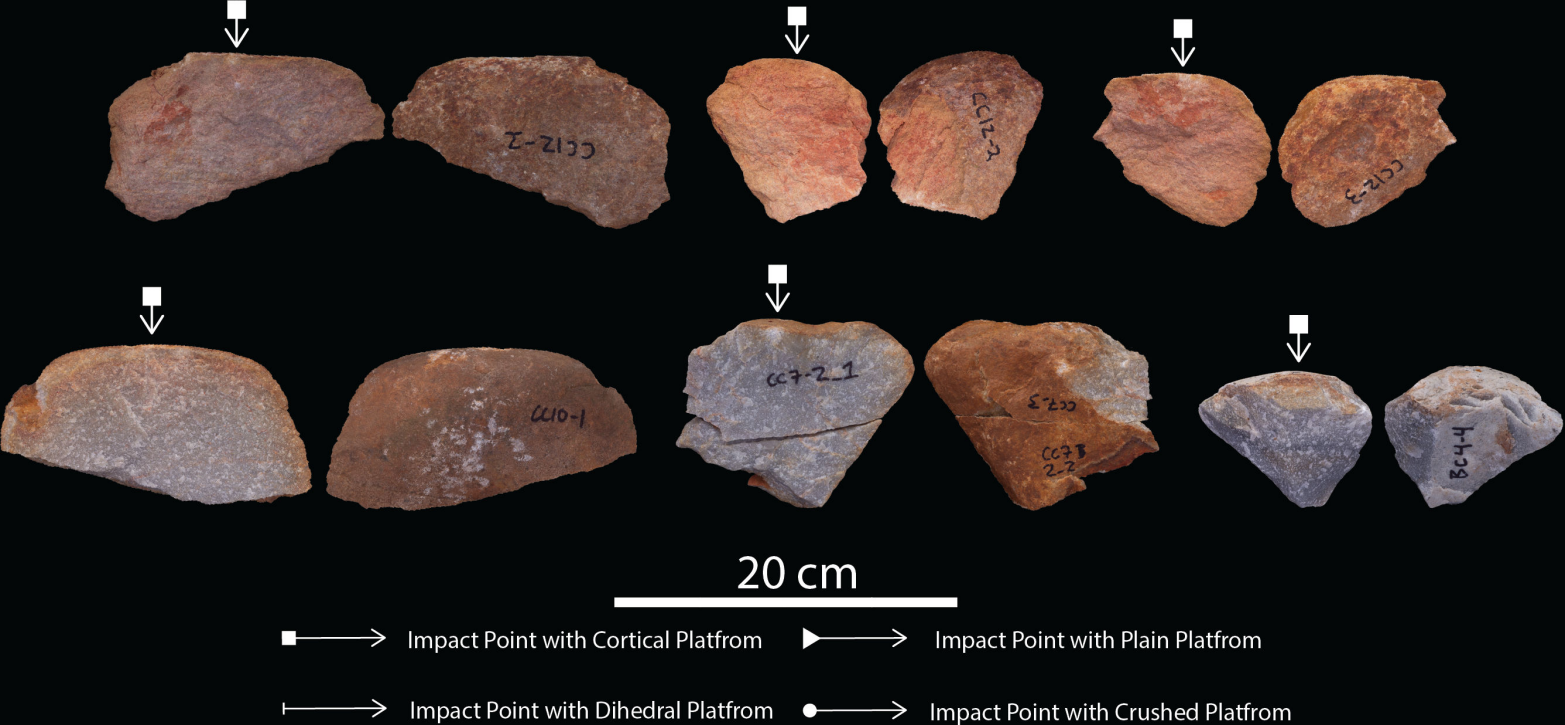
Novice large flake blanks detached from cores.

A total of 129 LFBs were produced from expert (*n* = 60), intermediate (*n* = 63) and novice (*n* = 6) skill groups (figures 6–8; table 1; electronic supplementary material, table S3). End-struck flakes, with the angle of detachment situated along the longest axis, are the most common blank type, with 42 (70%) of the expert, 42 (66%) of the intermediate and three (50%) of the novice samples (table 2; electronic supplementary material, table S3). Dorsal cortex is recorded on over >90% of flake blanks across each skill group (table 2). Longitudinal splits or transverse fractures in flake blank are recorded for 13 (22%) of the expert and 18 (28%) of the intermediate, and one (16%) of the novice samples (table 2; electronic supplementary material, table S3). Longitudinal splits generated at the point of percussion are the most common fracture type recorded across the expert and intermediate sample. Expert flakes averaged 1.5 dorsal flake scars per blank, compared with 1.3 for intermediate and 0.5 for novice blanks (table 2; electronic supplementary material, table S3). Dorsal scars on LFBs display a predominance for unidirectional patterns, with an increase in sub-radial and radial scar patterns in the expert sample (tables 2 and 3). Platform types are predominantly cortical or plain, however, platform maintenance is exhibited in 18% (*n* = 11) of the expert sample with dihedral platforms (figures 6–8; table 2; electronic supplementary material, table S3).

### 4.3. Efficiency and productivity

**Figure 9.**
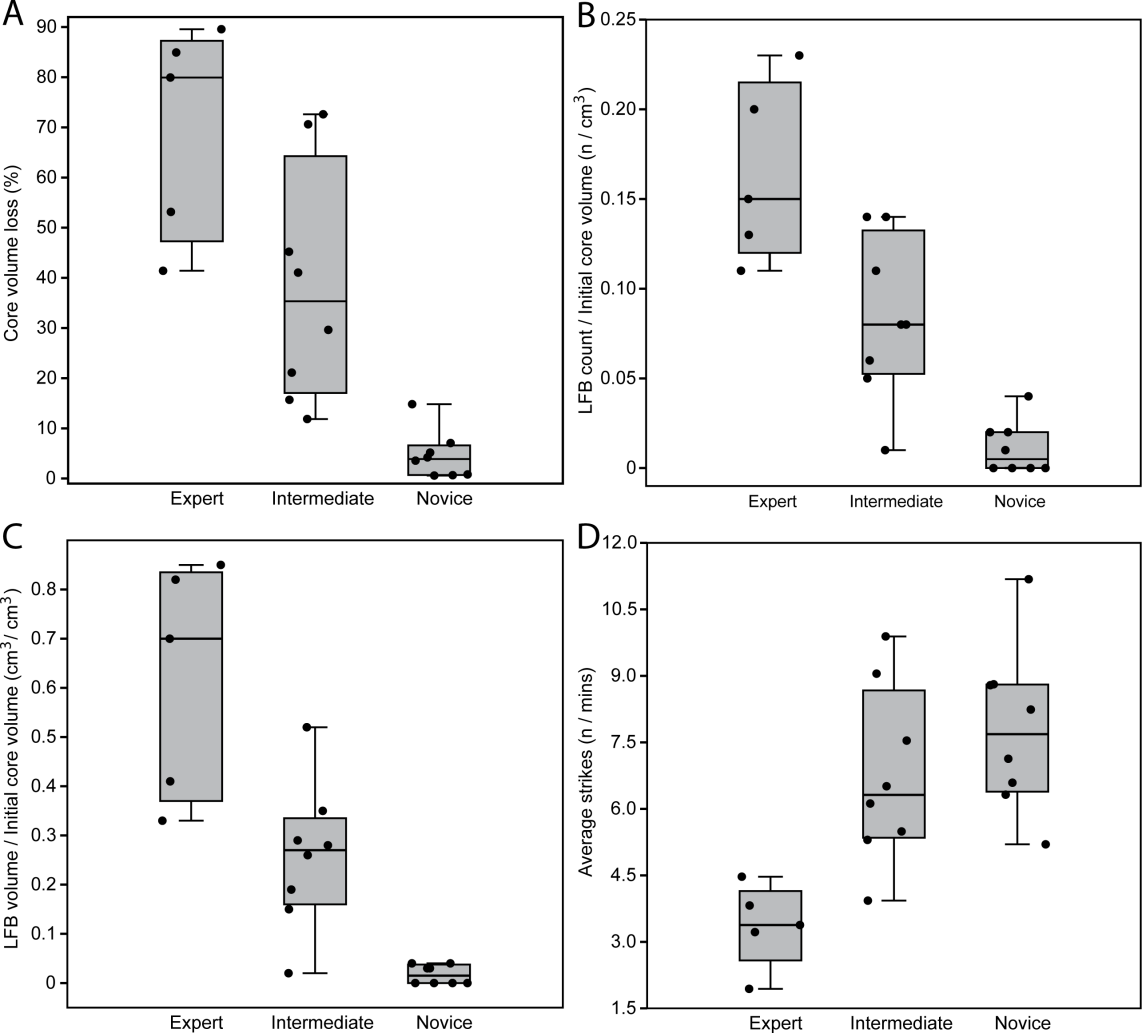
Boxplots comparing efficiency measures across skill groups. (A) The percentage of total core volume loss. (B) The ratio of LFBs to initial core volume. (C) The ratio of LFB volume to initial core volume. (D) The average number of strikes relative to total reduction time.

**Figure 10.**
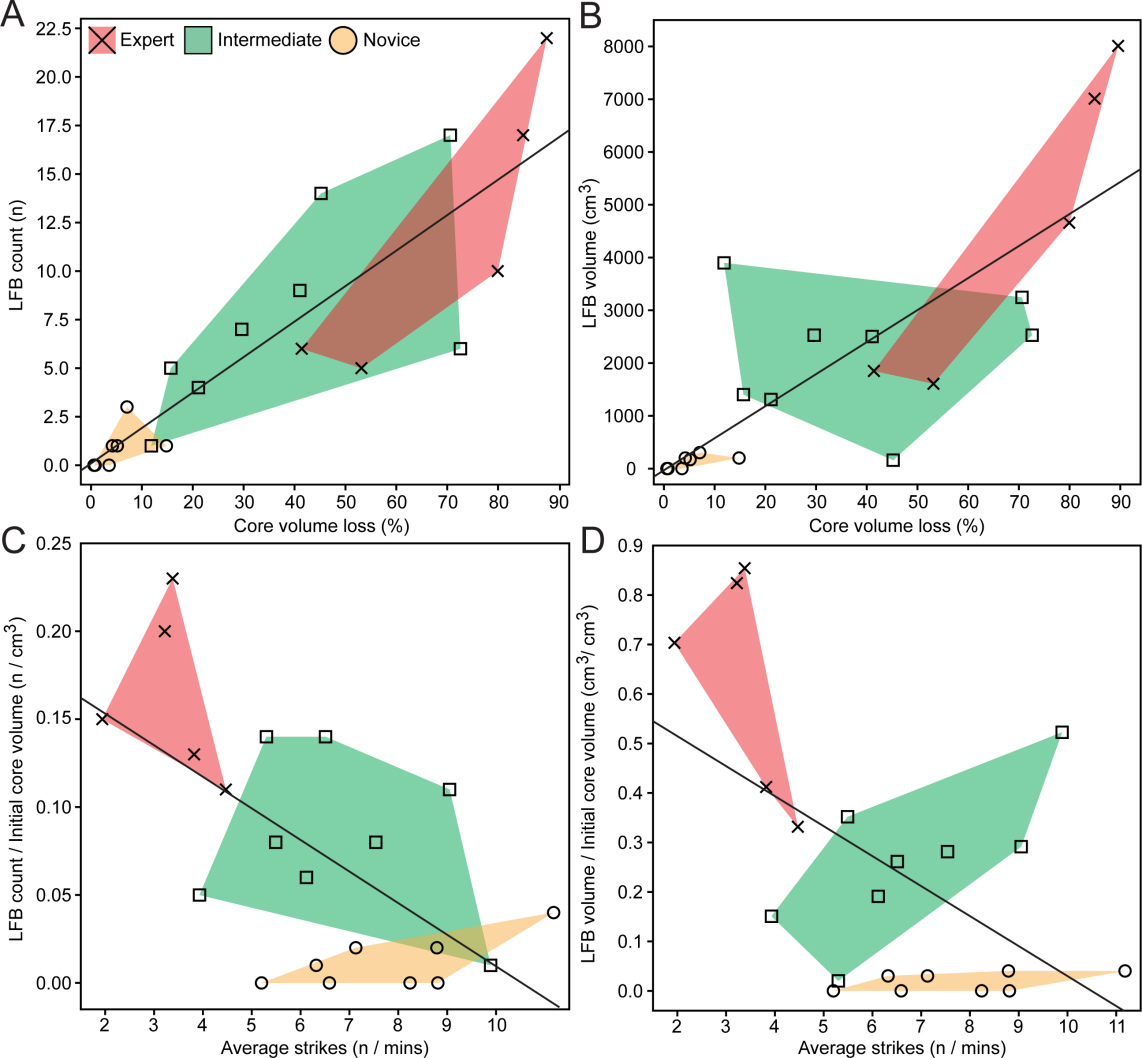
Regression graph comparing efficiency and productivity variables. (A) The percentage of core volume loss relative to LFB count. (B) The percentage of core volume loss relative to LFB volume. (C) The average number of strikes relative to the ratio of LFBs to initial core volume. (D) The average number of strikes relative to the ratio of LFB volume to initial core volume.

The results of Mann–Whitney *U* test results for all variables compared are reported in electronic supplementary material (see electronic supplementary material, tables S5–S7), which generally support significant differences in core reduction efficiency and large flake productivity across skill groups. The percentage of GC volume loss was significant between all skill groups, which shows that the expert reduced cores more intensely when compared to intermediates (*U* = 5, *z* = −2.19, *p* = 0.031) and novices (*U* = 0, *z* = −2.92, *p* = 0.001) (figure 9A; electronic supplementary material, table S5). The number of LFBs produced relative to initial core volume also shows significant differences between all skill groups (expert–intermediate: *U* = 4, *z* = −2.34, *p* = 0.017; expert–novice: *U* = 0, *z* = −2.96, *p* = 0.002; intermediate–novice: *U* = 4, *z* = −2.96, *p* = 0.002), suggesting that the conversion of core volume into LFBs increases proportionally with higher skill levels (figure 9B; electronic supplementary material, table S6). This trend is further confirmed by total LFB volume relative to initial core volume (expert–intermediate: *U* = 3, *z* = −2.48, *p* = 0.011; expert–novice: *U* = 0, *z* = −2.97, *p* < 0.001; intermediate–novice: *U* = 4, *z* = −2.96, *p* = 0.002), which is also significantly different across all skill groups (figure 9C; electronic supplementary material, table S6). Moreover, the average number of strikes is significantly different across all skill groups (expert–intermediate: *U* = 1, *z* = −2.78, *p* = 0.003; expert–novice: *U* = 1, *z* = −2.92, *p* = 0.002; intermediate–novice: *U* = 23, *z* = −0.94, *p* = 0.002), with the expert demonstrating the lowest number of strikes required to detach both debitage flakes and LFBs (figure 9D; electronic supplementary material, table S6). To compare levels of efficiency in GC reduction, the percentage of GC volume loss and average number of strikes were compared to both LFB count and volume relative to initial core volume in regression scatterplots (figure 10). The percentage of core volume loss relative to LFB counts and volume represents the successful conversion of core volume into blank products, which demonstrates that skilled performance is characterized by increased volume converted into LFBs (figure 10A,B). Moreover, the average number of strikes relative to the number of LFBs to initial core volume, as well as LFB volume to initial core volume, show an inverse relationship that highlights the effects of skill as lower energy costs required to convert higher amounts of core volume into LFBs (figure 10C,D).

**Figure 11.**
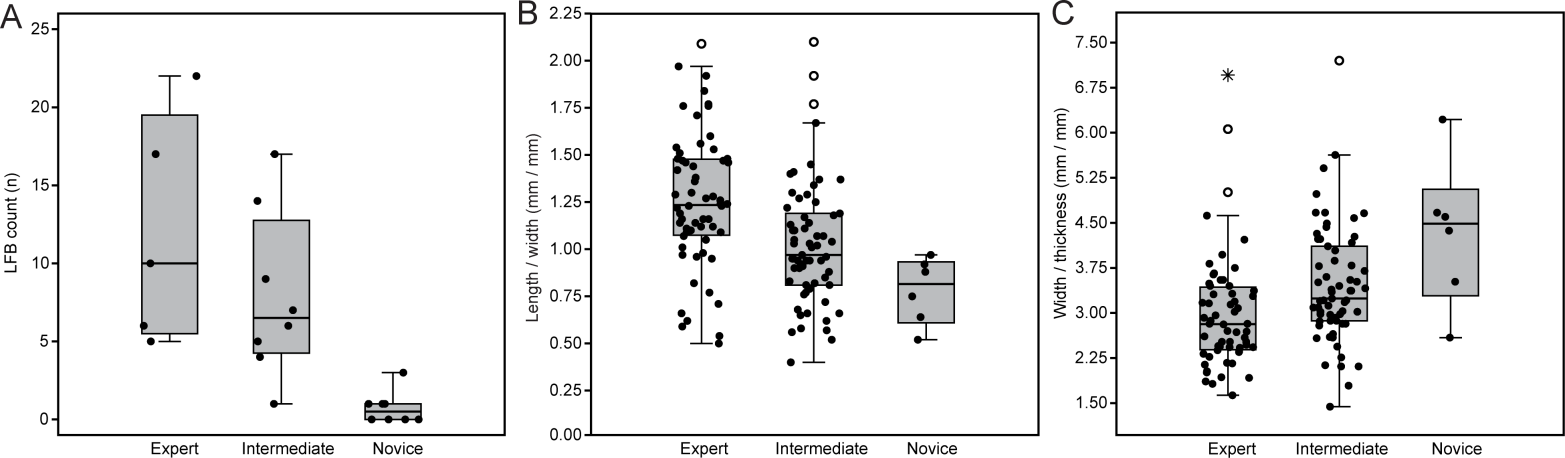
Boxplots comparing productivity measures across skill groups. (A) The total number of large flake blanks produced. (B) The ratio of length to width (elongation) in large flake blanks. (C) The ratio of width to thickness (refinement) in large flake blanks.

Productivity measures also demonstrate that expert performance yielded a higher number of LFBs with features amenable to LCT reduction. The number of LFBs produced only statistically differed between expert and novices (*U* = 0, *z* = −2.98, *p* = 0.001), as well as intermediates and novices (*U* = 2.5, *z* = −3.14, *p* = 0.001), whereas expert and intermediate produced a relatively similar amount, albeit through higher energy expenditure in intermediates (figure 11A; electronic supplementary material, table S5). Moreover, both elongation (L/W) and refinement (W/Th) ratios show significant differences between expert and the other skill groups (L/W expert–intermediate: *U* = 1104.5, *z* = −3.97, *p* < 0.001; W/Th expert–intermediate: *U* = 1244.5, *z* = −3.26, *p* = 0.001; L/W expert–novice: *U* = 37.5, *z* = −3.17, *p* < 0.001; W/Th expert–novice: *U* = 59.5, *z* = −2.68, *p* = 0.004), while only elongation of LFBs differed between intermediates and novices (*U* = 93, *z* = −2.04, *p* = 0.038) (figure 11B,C; electronic supplementary material, table S7). This suggests that intermediates and experts generally produce a higher number of elongated and thin LFBs, which are ideal for LCT shaping. The morphometric dimensions of blanks produced through skilled reduction thus provide appropriate surface area for managing the shape of LCT forms throughout reduction, as well as lessen the need for extensive thinning routines (cf. [4,13,15,18,22]).

## 5. Discussion

### 5.1. The role of technological skill in giant core reduction

The reduction strategies recorded in our experiments reveal important differences when comparing expert, intermediate and novice performances. The expert consistently prepared flaking surface convexities through the management of scar ridges and external platform angles, before choosing appropriate platforms to be prepared before striking, increasing the probability of producing elongated LFBs (figure 11; table 1; electronic supplementary material, table S3) [17]. The expert further rotated cores systematically and changed hammerstones according to the anticipated amount of force needed to detach flakes. These actions demonstrate a high degree of knowledge and flexibility in technical gestures, which increased the exploitation of core volumes, as well as the number of LFBs produced per core reduced. Difficulties were also encountered, including areas of lithological inconsistencies and the development of step fractures on flaking surfaces. However, the expert was able to overcome them through preparation, evaluating the natural geometry of GCs as reduction progressed and adjusting flaking sequences to remove or isolate step/hinge fractures.

Intermediate participants applied knapping strategies best suited to natural geometry of boulders, although propagated step/hinge fractures early in reduction sequences. An inability to prepare platforms and alter external flaking angles in the initial stages of reduction hindered the exploitation of core volumes. The intermediates also expended significant amounts of energy trying to strike through flaking surfaces with substantial step fractures, which compounded platform crushing (figures 3 and 5). As reduction progressed, edge angles and core surface convexities become unsuitable for LFB detachments. Furthermore, novice performance was characterized by inaccurate and repetitive percussive striking with little to no outcome in terms of LFB production. For example, one novice participant struck at the core edge of BCC-5 a total of 34 times before switching the core’s position and flaking strategy. Novice participants also constantly shifted and repositioned themselves, GCs and hammerstone grips throughout reduction sequences. The lack of stabilizing cores also decreased the accuracy of percussive strikes and force transfer, which resulted in high rates of platform crushing and step/hinge fractures (figure 5B,C). As a result, novice core reduction sequences were overall very short and primarily focused on a single edge or corner of a GC, with little awareness for core position and striking angles. Core volumes and LFB outputs were also comparatively low (electronic supplementary material, table S1). Maximizing blank outputs in GC reduction therefore requires expertise to overcome the challenges of LFB production and outweigh energetic costs. In Acheulian contexts, expert GC reduction may have also had important implications for economizing LCT manufacturing chains, which are highlighted in measures of efficiency and productivity.

### 5.2. Efficiency and productivity in large flake blank production

Our results suggest that expertise in GC reduction increases the efficiency of core-to-blank volume conversion and the production of standardized LFBs, while limiting energy expenditure. The ability to generate LFBs with morphological features amenable to shaping LCTs (i.e. elongated and thin) provides flexible pathways of manufacturing LCTs. Front-loaded time investment in creating LFBs reduces the technical sequences needed to shape and thin LCTs, which require high levels of technological skill [13,18–20,32]. As noted above, past experimental and archaeological research has highlighted the difficulties of handaxe thinning routines, which often require platform isolation and preparation, raising planes of bifacial intersections and managing the convexity of cross-sectional shapes [13,15,18–22,41,77,78]. Thinning phases of handaxe manufacturing is also associated with a high frequency of step and hinge fracturing, often related to weak percussive force transmission [18,19,41]. The investment in GC reduction and blank use likely mitigated much of these skill-dependent concerns in lessening the technical demands of LCT shaping and thinning [4,13,17]. The increase in LFB use after approximately 1 Ma, may therefore have mitigated skill-dependent, challenging phases of LCT manufacturing chains [4,11,12,15,16].

In support of this view, Wynn & Gowlett [16] summarized the ‘design imperatives’ of handaxe production, in which they acknowledge advantages of LFB use. They argued that handaxes are governed by specific design features including forward and lateral extensions in planview shape to support elongated forms, an extended, bifacial edge, as well as thickness adjustments to achieve relatively thin cross sections. They also suggested that LFBs allowed Acheulian knappers to achieve these design criteria with minimal, subsequent modification, thus inferring economic benefits embedded in GC reduction strategies (cf. [3,4,11,12]). Moreover, cleaver design was often dependent upon GC reduction, specifically in defining the bit, or the working edge, through scar ridges on core surfaces prior to their detachment as large flakes [30,87]. As such, GC reduction in LCT manufacturing potentially reflects an aspect of economization in Acheulian lithic production systems [12,13].

## 6. Conclusion

Beyond the benefits of LCT manufacture, the rise of formalized GC reduction strategies further reveals a layer of planning depth in Acheulian technological behaviours. In fact, standardized LFB production and use may have had wide-ranging effects on the organization of LCT manufacturing, including associated social and cognitive scaffolding. Petraglia *et al.* [88] investigated the spatial structure of the Acheulian quarry at Isampur in the Hunsgi-Baichbal Valley in India, finding that the logistics of acquiring hammerstones and boulders in the preparatory phases of LCT production reflected a structured use of the landscape based on planned exploitation of lithic resources. Similar conclusions were reached concerning the occupation of Amanzi Springs Areas 1 and 2 along the southern coast of South Africa, where the use of these localities was likely based on the availability of boulders and cobbles for lithic production, along with unique faunal and floral resources [58,59]. Further drawing from ethnographic accounts of skill acquisition in lithic production systems, technological expertise would have greatly benefited the planning and resource acquisition phases of Acheulian lithic manufacture to limit unnecessary energy expenditure and maximize the efficiency of blank production (cf. [35,36,61,89]).

Studies focused on the social organization of lithic manufacturing have argued that observation and imitation likely played a key role in learning the phases of LCT production [90–92]. In this context, highly skilled GC reduction may have been important for generating standardized blank forms that knappers of varying skill levels could use to shape LCTs. The ability to create quantities of standardized blanks may have aided the training of neophyte knappers, where experts could potentially guide novices and intermediates in acquiring both knowledge and experience (cf. [35,36,62]; also see [12,15]). While it is difficult to detect signs of apprenticeship in the Acheulian archaeological record, some possible evidence comes from Qesem Cave in Israel, where cores bearing the stigma of novice knapping performance, e.g. platform edge battering and pervasive step and hinge fractures, were seemingly, initially shaped by individuals with higher levels of technological skill for the purpose of training [62,93]. Such interpretations exemplify the importance of shaping blanks when training, which in turn improves the number of skilled tool producers within groups, over time.

Lastly, flexibility in LCT manufacturing chains has implications for cognitive capacities that structure associated behaviours. Both Herzlinger *et al.* [30] and Alperson-Afil *et al.* [47] emphasized the role of expert cognition in the LCT production and subsistence behaviours documented at Gesher Benot Ya’aqov in Israel, based on the reconstruction of complex technical sequences underlying such tasks. With respect to cleaver production at this site, Herlzinger *et al.* [30] argued that the preparation of flake scar patterns on GCs used to create cleaver bits was guided by highly skilled actions. The production of Kombewa flakes, as a distinct category of cleaver type also required complex reduction sequences to achieve desired working edge morphologies. These technical sequences demonstrate a clear ‘tool concept’ that could be achieved through multiple reduction procedures. As such, hierarchically structured procedural units guiding LCT production likely relied on working memory functions that facilitated the recall of associated procedural memories. Moreover, these capacities allowed Acheulian knappers to reorder specific phases of LCT manufacturing chains that mitigated the skill-dependent demands of extensive shaping and thinning [30]. We argue that GC reduction in the Acheulian archaeological record also reflects expert cognition, where anticipatory planning of both resource acquisition (i.e. hammerstones and boulders) and the application of specific reduction trajectories required expert retrieval capacities to successfully produce standardized blanks efficiently.

## Data Availability

The authors confirm that the data supporting the findings of this study are available within the article and/or its supplementary materials [94].

## References

[1] Sharon G. 2007 Acheulian large flake industries: technology, chronology, and significance. Oxford, U.K: BAR International Series.

[2] Sharon G. 2009 Acheulian giant-core technology: a worldwide perspective. Curr. Anthropol. **50**, 335–367. (10.1086/598849)

[3] Sharon G. 2011 Flakes crossing the straits? Entame flakes and northern Africa–Iberia contact during the Acheulean. Afr. Archaeol. Rev. **28**, 125–140. (10.1007/s10437-011-9087-3)

[4] Sharon G. 2010 Large flake Acheulian. Quat. Int. **223**, 226–233. (10.1016/j.quaint.2009.11.023)

[5] Paddayya K, Jhaldiyal R, Petraglia MD. 2006 The Acheulian quarry at Isampur, lower Deccan, India. In Axe age: Acheulean tool making from quarry to discard (eds N Goren-Inbar, G Sharon), pp. 45–73. London, UK: Equinox.

[6] Mishra S, Gaillard C, Deo S, Singh M, Abbas R, Agrawal N. 2010 Large flake Acheulian in India: implications for understanding lower Pleistocene human dispersals. Quat. Int. **223**, 271–272. (10.1016/j.quaint.2009.11.005)

[7] Shipton C, Parton A, Breeze P, Jennings RP, Groucutt HS, White TS. 2014 Large flake Acheulean in the Nefud Desert of northern Arabia. PaleoAnthropology **2014**, 446–462.

[8] Rubio-Jara S, Panera J, Rodríguez-de-Tembleque J, Santonja M, Pérez-González A. 2016 Large flake Acheulean in the middle of Tagus basin (Spain): middle stretch of the river Tagus valley and lower stretches of the rivers Jarama and Manzanares valleys. Quat. Int. **411**, 349–366. (10.1016/j.quaint.2015.12.023)

[9] Li H, Kuman K, Lotter MG, Leader GM, Gibbon RJ. 2017 The Victoria West: earliest prepared core technology in the Acheulean at Canteen Kopje and implications for the cognitive evolution of early hominids. R. Soc. Open Sci. **4**, 170288. (10.1098/rsos.170288) 28680682 PMC5493924

[10] Li H, Kuman K, Leader GM, Couzens R. 2018 Handaxes in South Africa: two case studies in the early and later Acheulean. Quat. Int. **480**, 29–42. (10.1016/j.quaint.2016.08.025)

[11] Baena Preysler J, Torres Navas C, Sharon G. 2018 Life history of a large flake biface. Quat. Sci. Rev. **190**, 123–136. (10.1016/j.quascirev.2018.04.015)

[12] Wilson CG, Caruana MV, Blackwood AF, Arnold LJ, Herries AIR. 2024 Why large flakes? Later Acheulian handaxe manufacture at amanzi springs, area 2 (Eastern Cape, South Africa). J. Archaeol. Sci. **53**, 104393. (10.1016/j.jasrep.2024.104393)

[13] Winton V. 2005 An investigation of knapping‐skill development in the manufacture of Palaeolithic handaxes. In Stone knapping: the necessary conditions for a uniquely hominin behaviour (eds V Roux, B Bril), pp. 109–116. Cambridge, UK: McDonald Institute for Archaeological Research.

[14] Shipton C. 2018 Biface knapping skill in the East African Acheulean: progressive trends and random walks. Afr. Archaeol. Rev. **35**, 107–131. (10.1007/s10437-018-9287-1)

[15] Shipton C. 2022 Predetermined refinement: the earliest Levallois of the Kapthurin Formation. J. Paleolit. Archaeol. **5**, 4. (10.1007/s41982-021-00109-1)

[16] Wynn T, Gowlett J. 2018 The handaxe reconsidered. Evol. Anthropol. **27**, 21–29. (10.1002/evan.21552) 29446559

[17] Wilson CG, Caruana MV, Bradley B, Muir RA, Blackwood AF, Herries AIR. 2024 An actualistic experimental study of giant quartzite core reduction strategies: implications for large flake blank production and handaxe manufacture at Amanzi Springs, South Africa. J. Field Archaeol. 1–17. (10.1080/00934690.2024.2401284)

[18] Callahan E. 1979 Basics of biface knapping in the eastern fluted point tradition: a manual for flintknappers and lithic analysts. Archaeol. East N. Am. **7**, 1–180.

[19] Edwards SW. 2001 A modern Knapper’s assessment of the technical skills of the Late Acheulean biface workers at Kalambo Falls. In Kalambo falls prehistoric site. the earlier cultures: middle and earlier stone age (ed. JD Clark), pp. 605–611, vol. III. Cambridge, UK: Cambridge University Press.

[20] Stout D, Apel J, Commander J, Roberts M. 2014 Late Acheulean technology and cognition at Boxgrove, UK. J. Archaeol. Sci. **41**, 576–590. (10.1016/j.jas.2013.10.001)

[21] Caruana MV. 2021 Pilot study comparing the effects of thinning processes on the cross‐sectional morphologies of early and late Acheulian handaxes. Archaeometry **63**, 481–499. (10.1111/arcm.12635)

[22] Caruana MV, Herries AIR. 2021 Modelling production mishaps in later Acheulian handaxes from the area 1 excavation at Amanzi Springs (Eastern Cape, South Africa) and their effects on reduction and morphology. J. Archaeol. Sci. **39**, 103121. (10.1016/j.jasrep.2021.103121)

[23] Sharon G, Beaumont P. 2006 Victoria west–a highly standardized prepared core technology. In Axe age: Acheulean tool-making from quarry to discard (eds N Goren-Inbar, G Sharon), pp. 181–199. London, UK: Equinox.

[24] Goren-Inbar N. 2011 Culture and cognition in the Acheulian industry: a case study from Gesher Benot Yaʿaqov. Phil. Trans. R. Soc. B Biol. Sci. **366**, 1038–1049. (10.1098/rstb.2010.0365) PMC3049101 21357226

[25] Jones PR. 1994 Results of experimental work in relation to the stone industries of Olduvai Gorge. In Olduvai Gorge: volume 5, excavations in beds III, IV and the Masek beds (ed. M Leakey), pp. 254–298. Cambridge, UK: Cambridge University Press.

[26] Toth N. 2001 Experiments in quarrying large flake blanks at Kalambo Falls. In Kalambo falls prehistoric site (ed. JD Clark), pp. 600–604, vol. III. Cambridge, UK: Cambridge University Press.

[27] Madsen B, Goren-Inbar N. 2004 Acheulian giant core technology and beyond: an archaeological and experimental case study. Eurasian Prehistory **2**, 3–52.

[28] Goren-Inbar N, Grosman L, Sharon G. 2011 The technology and significance of the Acheulian giant cores of Gesher Benot Ya‘aqov, Israel. J. Archaeol. Sci. **38**, 1901–1917. (10.1016/j.jas.2011.03.037)

[29] Stout D. 2011 Stone toolmaking and the evolution of human culture and cognition. Phil. Trans. R. Soc. B Biol. Sci. **366**, 1050–1059. (10.1098/rstb.2010.0369) PMC3049103 21357227

[30] Herzlinger G, Wynn T, Goren-Inbar N. 2017 Expert cognition in the production sequence of Acheulian cleavers at Gesher Benot Ya’aqov, Israel: a lithic and cognitive analysis. PLoS One **12**, e0188337. (10.1371/journal.pone.0188337) 29145489 PMC5690685

[31] Herzlinger G, Goren-Inbar N. 2019 Do a few tools necessarily mean a few people? A techno-morphological approach to the question of group size at Gesher Benot Ya’aqov, Israel. J. Hum. Evol. **128**, 45–58. (10.1016/j.jhevol.2018.11.008) 30825981

[32] Pargeter J, Khreisheh N, Stout D. 2019 Understanding stone tool-making skill acquisition: experimental methods and evolutionary implications. J. Hum. Evol. **133**, 146–166. (10.1016/j.jhevol.2019.05.010) 31358178

[33] Pargeter J, Khreisheh N, Shea JJ, Stout D. 2020 Knowledge vs. know-how? Dissecting the foundations of stone knapping skill. J. Hum. Evol. **145**, 102807. (10.1016/j.jhevol.2020.102807) 32485326

[34] Liu C, Khreisheh N, Stout D, Pargeter J. 2023 Differential effects of knapping skill acquisition on the cultural reproduction of Late Acheulean handaxe morphology: archaeological and experimental insights. J. Archaeol. Sci. **49**, 103974. (10.1016/j.jasrep.2023.103974)

[35] Stout D. 2002 Skill and cognition in stone tool production. Curr. Anthropol. **43**, 693–722. (10.1086/342638)

[36] Stout D. 2005 The social and cultural context of stone-knapping skill acquisition. In Stone knapping: the necessary conditions for a uniquely hominin behaviour (eds V Roux, B Bril), pp. 331–340. Cambridge, UK: McDonald Institute for Archaeological Research.

[37] Putt SS. 2015 The origins of stone tool reduction and the transition to knapping: an experimental approach. J. Archaeol. Sci. **2**, 51–60. (10.1016/j.jasrep.2015.01.004)

[38] Muller A, Clarkson C. 2016 Identifying major transitions in the evolution of lithic cutting edge production rates. PLoS One **11**, e0167244. (10.1371/journal.pone.0167244) 27936135 PMC5147885

[39] Muller A, Shipton C, Clarkson C. 2022 Stone toolmaking difficulty and the evolution of hominin technological skills. Sci. Rep. **12**, 5883. (10.1038/s41598-022-09914-2) 35393496 PMC8989887

[40] Režek Ž, Dibble HL, McPherron SP, Braun DR, Lin SC. 2018 Two million years of flaking stone and the evolutionary efficiency of stone tool technology. Nat. Ecol. Evol. **2**, 628–633. (10.1038/s41559-018-0488-4) 29507377

[41] Shelley PH. 1990 Variation in lithic assemblages: an experiment. J. Field Archaeol. **17**, 187–193. (10.1179/009346990791548349)

[42] Eren MI, Bradley BA, Sampson CG. 2011 Middle Paleolithic skill level and the individual knapper: an experiment. Am. Antiq. **76**, 229–251. (10.7183/0002-7316.76.2.229)

[43] Goldstein ST. 2019 Knowledge transmission through the lens of lithic production: a case study from the Pastoral Neolithic of southern Kenya. J. Archaeol. Method Theory **26**, 679–713. (10.1007/s10816-018-9387-x)

[44] Pargeter J, de la Peña P, Eren MI. 2019 Assessing raw material’s role in bipolar and freehand miniaturized flake shape, technological structure, and fragmentation rates. Archaeol. Anthropol. Sci. **11**, 5893–5907. (10.1007/s12520-018-0647-1)

[45] Wilson EP, Stout D, Liu C, Kilgore MB, Pargeter J. 2023 Skill and core uniformity: an experiment with Oldowan-like flaking systems. Lithic Technol. **48**, 333–346. (10.1080/01977261.2023.2178767)

[46] Muller A, Clarkson C. 2023 Filling in the blanks: standardization of lithic flake production throughout the stone age. Lithic Technol. **48**, 222–236. (10.1080/01977261.2022.2103290)

[47] Alperson-Afil N, Goren-Inbar N, Herzlinger G, Wynn T. 2020 Expert retrieval structures and prospective memory in the cognition of Acheulian hominins. Psychology **11**, 173–189. (10.4236/psych.2020.111012)

[48] Wynn T, Haidle MN, Lombard M, Coolidge FL. 2017 The expert cognition model in human evolutionary studies. In Cognitive models in paleolithic archaeology (eds T Wynn, FL Coolidge), pp. 21–44. Oxford, UK: Oxford University Press.

[49] Wynn T, Coolidge FL. 2019 The role of expert technical cognition in human evolution. In Handbook of cognitive archaeology (eds TB Henley, MJ Rossano, EP Kardas), pp. 261–283. London, UK: Routledge Press. (10.4324/9780429488818-14)

[50] Beyene Y *et al*. 2013 The characteristics and chronology of the earliest Acheulean at Konso, Ethiopia. Proc. Natl Acad. Sci. USA **110**, 1584–1591. (10.1073/pnas.1221285110) 23359714 PMC3562807

[51] Diez-Martín F *et al*. 2015 The origin of the Acheulean: the 1.7 million-year-old site of FLK West, Olduvai Gorge (Tanzania). Sci. Rep. **5**, 17839. (10.1038/srep17839) 26639785 PMC4671088

[52] Gossa T, Hovers E. 2024 The emergence of large flake-based Acheulian technology: perspective from the highland site-complex of Melka Wakena, Ethiopia. Archaeol. Anthropol. Sci. **16**, 172. (10.1007/s12520-024-02072-8)

[53] Mussi M *et al*. 2023 Early Homo erectus lived at high altitudes and produced both Oldowan and Acheulean tools. Science **382**, 713–718. (10.1126/science.add9115) 37824630

[54] Diez-Martín F, Sánchez Yustos P, Gómez de la Rúa D, Gómez González JÁ, de Luque L, Barba R. 2014 Early Acheulean technology at Es2-Lepolosi (ancient MHS-Bayasi) in Peninj (Lake Natron, Tanzania). Quat. Int. **322**, 209–236. (10.1016/j.quaint.2013.08.053)

[55] Diez- Martín F, Sánchez Yustos P, Gómez González JÁ, Luque L, Gómez de la Rúa D, Domínguez-Rodrigo M. 2014 Reassessment of the early Acheulean at EN1-Noolchalai (ancient RHS-Mugulud) in Peninj (Lake Natron, Tanzania). Quat. Int. **322**, 237–263. (10.1016/j.quaint.2013.10.011)

[56] Méndez-Quintas E, Mussi M. 2025 Unravelling the development of large flake technology during the early Acheulean: the evidence from simbiro gully at melka Kunture (Upper Awash, Ethiopia). J. Paleo. Arch. **8**. (10.1007/s41982-024-00207-w)

[57] Sharon G. 2008 The impact of raw material on Acheulian large flake production. J. Archaeol. Sci. **35**, 1329–1344. (10.1016/j.jas.2007.09.004)

[58] Herries AIR *et al*. 2022 A marine isotope stage 11 coastal Acheulian workshop with associated wood at Amanzi Springs Area 1, South Africa. PLoS One **17**, e0273714. (10.1371/journal.pone.0273714) 36264956 PMC9584507

[59] Caruana MV, Wilson CG, Arnold LJ, Blackwood AF, Demuro M, Herries AIR. 2023 A marine isotope stage 13 Acheulian sequence from the Amanzi Springs Area 2 deep sounding excavation, Eastern Cape, South Africa. J. Hum. Evol. **176**, 103324. (10.1016/j.jhevol.2023.103324) 36812778

[60] Toth NP. 1982 The stone technologies of early hominids at Koobi Fora, Kenya: An experimental approach. PhD thesis, [Berkeley]: University of California.

[61] Holdaway S, Douglass M. 2012 A twenty-first century archaeology of stone artifacts. J. Archaeol. Method Theory **19**, 101–131. (10.1007/s10816-011-9103-6)

[62] Assaf E, Barkai R, Gopher A. 2016 Knowledge transmission and apprentice flint-knappers in the Acheulo-Yabrudian: a case study from Qesem Cave, Israel. Quat. Int. **398**, 70–85. (10.1016/j.quaint.2015.02.028)

[63] Eren MI, Greenspan A, Sampson CG. 2008 Are Upper Paleolithic blade cores more productive than Middle Paleolithic discoidal cores? A replication experiment. J. Hum. Evol. **55**, 952–961. (10.1016/j.jhevol.2008.07.009) 18835009

[64] Liu C, Stout D. 2023 Inferring cultural reproduction from lithic data: a critical review. Evol. Anthropol. **32**, 83–99. (10.1002/evan.21964) 36245296

[65] Maloney TR. 2019 Towards quantifying teaching and learning in prehistory using stone artifact reduction sequences. Lithic Technol. **44**, 36–51. (10.1080/01977261.2018.1564855)

[66] Perreault C, Brantingham PJ, Kuhn SL, Wurz S, Gao X. 2013 Measuring the complexity of lithic technology. Curr. Anthropol. **54**, S397–S406. (10.1086/673264)

[67] Eerkens JW, Bettinger RL. Techniques for assessing standardization in artifact assemblages: can we scale material variability? Am. Antiq. **66**, 493–504.

[68] Prasciunas MM. 2007 Bifacial cores and flake production efficiency: an experimental test of technological assumptions. Am. Antiq. **72**, 334–348. (10.2307/40035817)

[69] Jennings TA, Pevny CD, Dickens WA. 2010 A biface and blade core efficiency experiment: implications for early Paleoindian technological organization. J. Archaeol. Sci. **37**, 2155–2164. (10.1016/j.jas.2010.02.020)

[70] Clarkson C. 2013 Measuring core reduction using 3D flake scar density: a test case of changing core reduction at Klasies River Mouth, South Africa. J. Archaeol. Sci. **40**, 4348–4357. (10.1016/j.jas.2013.06.007)

[71] Johnson CL, Bolorbat T, Grote MN, Paine CH, Lkhundev G, Odsuren D, Izuho M, Gunchinsuren B, Zwyns N. 2024 Analyzing blank cutting edge efficiency associated with the adoption of microblade technology: a case study from Tolbor-17, Mongolia. PLoS One **19**, e0305136. (10.1371/journal.pone.0305136) 39150911 PMC11329150

[72] Pargeter J, Eren MI. 2017 Quantifying and comparing bipolar versus freehand flake morphologies, production currencies, and reduction energetics during lithic miniaturization. Lithic Technol. **42**, 90–108. (10.1080/01977261.2017.1345442)

[73] Pargeter J, Cebeiro A, Levy SB. 2024 Stone toolmaking energy expenditure differs between novice and expert toolmakers. Am. J. Biol. Anthropol. **185**, e25026. (10.1002/ajpa.25026) 39288016

[74] Shipton CBK, Petraglia MD, Paddayya K. 2009 Stone tool experiments and reduction methods at the Acheulean site of Isampur Quarry, India. Antiquity **83**, 769–785. (10.1017/s0003598x00098987)

[75] Li H, Li C, Sherwood NL, Kuman K. 2017 Experimental flaking in the Danjiangkou Reservoir Region (central China): a rare case of bipolar blanks in the Acheulean. J. Archaeol. Sci. **13**, 26–35. (10.1016/j.jasrep.2017.03.032)

[76] Wynn T, Coolidge FL. 2014 Technical cognition, working memory and creativity. Pragmat. Congn. **22**, 45–63. (10.1075/pc.22.1.03wyn)

[77] Shipton C, Clarkson C, Pal JN, Jones SC, Roberts RG, Harris C, Gupta MC, Ditchfield PW, Petraglia MD. 2013 Generativity, hierarchical action and recursion in the technology of the Acheulean to Middle Palaeolithic transition: a perspective from Patpara, the Son Valley, India. J. Hum. Evol. **65**, 93–108. (10.1016/j.jhevol.2013.03.007) 23746433

[78] Shipton C. 2023 Recursive narrative and the Acheulean to Middle Palaeolithic transition. In Oxford handbook of cognitive archaeology (eds T Wynn, KA Overmann, FL Coolidge), pp. 723–740. Oxford, UK: Oxford University Press. (10.1093/oxfordhb/9780192895950.013.31)

[79] Goren-Inbar N, Alperson-Afil N, Sharon G, Herzlinger G. 2018 The acheulian site of gesher benot ya ‘Aqov volume iv: the lithic assemblages. Cham, Switzerland: Springer.

[80] Roe DA. 1969 British Lower and Middle Palaeolithic handaxe groups. Proc. Prehist. Soc. **34**, 1–82. (10.1017/s0079497x00013840)

[81] Isaac GL, Keller CM. 1968 Note on the proportional frequency of side- and end-struck flakes. South Afr. Archaeol. Bull. **23**, 17. (10.2307/3887686)

[82] Andrefsky W. 2005 Lithics: macroscopic approaches to analysis. Cambridge, UK: Cambridge University Press.

[83] Holdaway SJ, Stern N. 2004 A record in stone: the study of Australia’s flaked stone artifacts. Melbourne, Australia: Melbourne Museum Victoria and Aboriginal Studies Press.

[84] Geribàs N, Mosquera M, Vergès JM. 2010 What novice knappers have to learn to become expert stone toolmakers. J. Archaeol. Sci. **37**, 2857–2870.

[85] Diez-Martín F, Cuartero F, Yustos PS, Baena J, Rubio D, Domínguez-Rodrigo M. 2012 Testing cognitive skills in early Pleistocene hominins: an analysis of the concepts of hierarchization and predetermination in the lithic assemblages of TypeSection (Peninj, Tanzania). In Stone tools and fossil bones: debates in the archaeology of human origins (ed. M Dominguez-Rodrigo), pp. 245–309. Cambridge, UK: Cambridge University Press. (10.1017/CBO9781139149327.012)

[86] Sánchez‐Yustos P, Diez‐Martín F, Domínguez‐Rodrigo M, Duque J, Fraile C, Baquedano E, Mabulla A. 2017 Diversity and significance of core preparation in the developed Oldowan technology: reconstructing the flaking processes at SHK and BK (Middle‐Upper Bed II, Olduvai Gorge, Tanzania). Boreas **46**, 874–893. (10.1111/bor.12237)

[87] Sharon G. 2019 Early convergent cultural evolution. In Squeezing minds from stones (eds KA Overmann, FL Coolidge), pp. 237–250. Oxford, UK: Oxford University Press. (10.1093/oso/9780190854614.003.0012)

[88] Petraglia MD, Shipton C, Paddayya K. 2005 Life and mind in the Acheulean: a case study from India. In Hominid individual in context: investigations of lower and Middle Palaeolithic landscapes, locales and artefacts (eds C Gamble, M Porr), pp. 197–219. London: Routledge.

[89] Roux V, Bril B, Dietrich G. 1995 Skills and learning difficulties involved in stone knapping: the case of stone‐bead knapping in Khambhat, India. World Archaeol. **27**, 1. (10.1080/00438243.1995.9980293)

[90] Shipton C. 2010 Imitation and shared intentionality in the Acheulean. Camb. Archaeol. J. **20**, 197–210. (10.1017/s0959774310000235)

[91] Lycett SJ, Schillinger K, Kempe M, Mesoudi A. 2015 Learning in the Acheulean: experimental insights using handaxe form as a ‘model organism. In Learning strategies and cultural evolution during the palaeolithic (eds A Mesoudi, K Aoki), pp. 155–166. New York, NY, USA: Springer. (10.1007/978-4-431-55363-2_11)

[92] Schillinger K, Mesoudi A, Lycett SJ. 2015 The impact of imitative versus emulative learning mechanisms on artifactual variation: implications for the evolution of material culture. Evol. Hum. Behav. **36**, 446–455. (10.1016/j.evolhumbehav.2015.04.003)

[93] Assaf E. 2017 Core sharing: the transmission of knowledge of stone tool knapping in the Lower Palaeolithic, Qesem Cave (Israel). Hunt. Gatherer Res **3**, 367–399. (10.3828/hgr.2017.19)

[94] Wilson C, Caruana M, Bradley B, Herries A. 2025 Supplementary material from: Skill and Efficiency in Acheulian Giant Core Reduction. FigShare. (10.6084/m9.figshare.c.7885853) PMC12187401 40568557

